# STAT-ART: The Promise and Practice of a Rapid Palliative Single Session of MR-Guided Online Adaptive Radiotherapy (ART)

**DOI:** 10.3389/fonc.2019.01013

**Published:** 2019-10-22

**Authors:** Kathryn E. Mittauer, Patrick M. Hill, Mark W. Geurts, Anna-Maria De Costa, Randall J. Kimple, Michael F. Bassetti, John E. Bayouth

**Affiliations:** ^1^Department of Human Oncology, UW Carbone Cancer Center, University of Wisconsin-Madison, Madison, WI, United States; ^2^Department of Radiation Oncology, Baptist Health South Florida, Miami Cancer Institute, Miami, FL, United States; ^3^Department of Radiation Oncology, Aspirus Wausau Hospital, Aspirus Inc., Wausau, WI, United States

**Keywords:** online adaptive radiotherapy, MRgoART, MR-guidance, MRgRT, ART, MR-guided radiotherapy, palliative radiation, deformable image registration

## Abstract

This work describes a novel application of MR-guided online adaptive radiotherapy (MRgoART) in the management of patients whom urgent palliative care is indicated using statum-adaptive radiotherapy (STAT-ART). The implementation of STAT-ART, as performed at our institution, is presented including a discussion of the advantages and limitations compared to the standard of care for palliative radiotherapy on conventional c-arm linacs. MR-based treatment planning techniques of STAT-ART for density overrides and deformable image registration (DIR) of diagnostic CT to the treatment MR are also addressed.

## Introduction

The American Cancer Society estimated that annually in 2019 there are 1.76 million new cases of cancer with 606,880 cancer deaths (1). Most cancer deaths are associated with a decreased quality of life and painful end-of-life, likely due to loco-regional or metastatic disease progression (2, 3). Palliative radiotherapy (RT) allows for the management of patients with advanced stage cancer. Palliative RT directly relieves obstructions, bleeding, and cancer-related pain for patients not a candidate for or responding to opioid medication (2–13).

As the sensitivity and accuracy of cancer detection and subsequently cancer treatments progressively improve, the life expectancy for cancer patients is steadily rising even with metastatic disease (3). Thus, the continued management of these patients is of great importance (3). Due to multiple steps in standard radiation planning processes, palliative treatment may take 3–7 days post-consultation before the first treatment fraction is performed (3). Improving the palliative RT workflow by utilizing advanced technology to offer rapid, same day treatment can reduce the pre-treatment time period, allowing for near-immediate pain-relief and an improved quality of life for these patients.

The MRIdian™ cobalt and more recently released MRIdian linac (ViewRay Inc., Cleveland, OH) is an MR-guided radiotherapy (MRgRT) platform that integrates magnetic resonance imaging (MRI), radiotherapy delivery, treatment planning, image registration, and treatment record and delivery into a single unit (14, 15). The integrated approach enables MR-guided online adaptive radiotherapy (MRgoART), where the care team designs and delivers a treatment plan based on patient anatomy and position at the time of treatment setup [Mittauer et al. (under review); (16, 17)]. MRgoART, or simply online adaptive radiotherapy (ART), offers the opportunity for rapid and accurate palliative online adaptive radiation therapy, i.e., statum-ART (STAT-ART).

The purpose of this work is to describe a potential paradigm change in the management of palliative care in radiotherapy using STAT-ART. The implementation of STAT-ART, as performed at our institution, is presented including a discussion of the advantages and limitations compared to the standard of care for palliative radiotherapy performed on conventional c-arm linacs. MR-based treatment planning techniques for density overrides and deformable image registration (DIR) of diagnostic CT to the treatment MR are also addressed.

## Conventional Linac Workflow For Urgent Palliative Treatment

Conventional radiotherapy workflow utilizes a serial-based process map. Tasks are performed through multiple applications and platforms (i.e., PACS, simulator system, image registration software, segmentation software, treatment planning system, treatment delivery system, quality assurance software, record, and verify system). Each step of the serial radiotherapy workflow is executed by a unique stakeholder (i.e., physician, physicist, dosimetrist, therapist).

**Figure 1:**
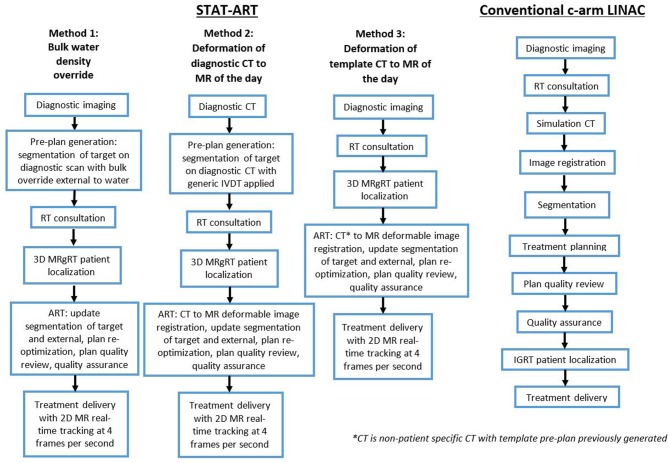
Workflow of three proposed methods of STAT-ART **(left)** compared to the standard of care for palliative treatment as performed on a conventional c-arm linac **(right)**.

The workflow process for conventional radiotherapy utilizing a c-arm linac will be briefly detailed here and is displayed in Figure 1. When a patient presents for initial consultation with their radiation oncologist, previously acquired diagnostic scans are reviewed at the time of consultation. Following consultation, the patient receives a CT simulation appointment in which a CT scan is acquired for purposes of treatment planning and treatment setup localization. Image registration of the diagnostic data to the planning CT scan is often performed to aid the physician in segmentation of the target and relevant surrounding anatomy. On the planning CT scan, the dosimetrist creates a treatment plan which subsequently undergoes plan quality review by the physician and physicist in addition to a quality assurance (QA) assessment. The patient is brought in for their treatment appointment, where x-ray based imaging is performed to localize the patient into the position as acquired at the time of the CT simulation, followed by radiotherapy treatment delivery.

To accelerate the radiotherapy process when a patient presents with urgent obstructions, bleeding, and/or pain, the above workflow is consolidated at the potential cost of the overall treatment plan quality. The treatment plan complexity is often limited for palliative cases to a parallel-opposed beam geometry with rectangular fields, defined by jaws alone rather than the use of more sophisticated multileaf collimation (MLC). Such plans are advantageous for simplifying other radiotherapy workflow processes such as simulation, setup, and treatment. However, utilizing more conformal planning and delivery techniques with more beam angles and more sophisticated collimation schemes can reduce dose to uninvolved organs and tissues.

As previously described, the standard of care utilizes a workflow of acquiring a CT simulation of the patient in setup position followed by a treatment planning session. The process may take several hours to several days. Additional steps to accelerate the process for urgent palliative radiotherapy include forgoing the simulation scan and performing 2D treatment planning based on 2D MV imaging on the radiotherapy treatment unit and calculation of dose to an arbitrary point within the patient. Without knowledge of spatial orientation of the tumor in treatment position, large target margins, and non-conformal dose distributions are required.

## STAT-ART Workflow For Urgent Palliative Treatment

### Overview of STAT-ART Workflow

The STAT-ART workflow takes advantage of the MRgoART features of the MRIdian to enable efficient adaptive planning capabilities and expedite the overall workflow [Mittauer et al. (under review); (16, 17)]. Prior to patient arrival and consultation, the generalized workflow (Figure 1) includes either generation of a patient-specific pre-plan or selection of a preexisting non-patient specific template plan. For the patient-specific pre-plan generation, the radiation oncologist performs segmentation of the target volume on the diagnostic dataset followed by the medical physicist or dosimetist performing the treatment planning (i.e., beam geometry, defining MLC aperture, beam weight optimization, and dose normalization). Details of the planning technique is described in the following sections.

**Figure 2:**
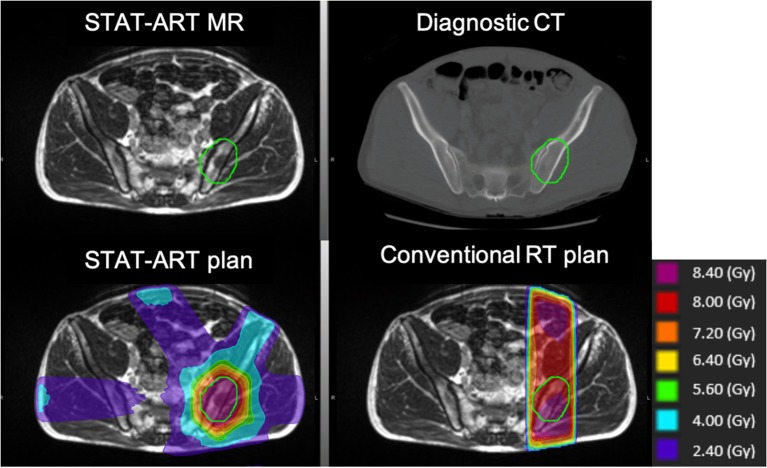
Clinical STAT-ART case of a pelvic bony metastasis treated to 8 Gy in a single fraction to the planning target volume in green. Comparison of diagnostic CT used for pre-planning (top right) and improved gross disease visualization on the TrueFISP treatment planning MR obtained using the MRIdian (top left). The resulting dose distribution for STAT-ART plan of a six-beam cobalt-60 dose distribution (bottom left) in comparison to a conventional plan with 10 MV AP/PA beams (bottom right). The STAT-ART plan shows a marked reduction of the high dose volume, particularly in anterior regions of normal tissues.

Following consultation, the patient is setup in an arbitrary treatment position and localized using and a 3D volumetric balanced steady-state free precession sequence (TrueFISP) MR scan (Figure 2) (18). A template-plan or pre-plan from the diagnostic scan is then adapted online based on the actual treatment geometry including updates to segmentation, beam apertures, optimization, and/or dose normalization. A plan quality visual inspection and calculation-based QA are performed for plan fidelity (17), followed by treatment delivery. The STAT-ART workflow will be dependent on institutional resources and MRgoART staffing model, and is generally executed in real-time by multiple staff members including a combination of therapist(s), a medical physicist, and a radiation oncologist.

### Planning Technique

Development of the STAT-ART protocol has emphasized efficiency. For the pre-plan generation, segmentation performed by the radiation oncologist is kept to a minimum with only two regions of interest requiring contouring: external or skin and a target volume. Initial planning of the pre-plan or template plan is performed by the medical physicist or dosimetrist with a single isocenter and 3D conformal beams defined by the MLC (19, 20). 3D conformal planning of the pre-plan or template plan involves defining an isocenter point of interest, inputting beams, setting gantry angles for ideal geometry, defining the MLC aperture, optimization of beam weights, and a Monte Carlo dose calculation with magnetic field corrections. As previously noted, online adaptive is then performed to make the corresponding plan adjustments to the patient's on table anatomy through segmentation updates to the target and corresponding plan MLC aperture shape and beam weight through plan re-optimization.

Typically, STAT-ART treatment plans use a six-beam arrangement, equivalent to two gantry positions for the three ^60^Co sources on the MRIdian cobalt. For the MRIdian linac, the higher dose rate has enabled comparable delivery times to the MRIdain cobalt even with an increase in gantry rotation required for the six-beam arrangement. Due to the utilization of 3D conformal planning technique, STAT-ART plans have similar delivery times to conventional AP/PA beam arrangements while allowing for more comparatively superior dose distributions (Figure 2).

### Electron Density for MR-Based Planning

MR-based planning requires electron density information for dose calculation purposes. We present three strategies (Figure 1) in the STAT-ART workflow for density propagation using bulk density overrides or deformable image registration for respective homogenous or heterogenous dose calculations.

#### Bulk Density Override

For non-thoracic based anatomical sites, a single bulk density override of the external region of interest to water can be used for electron density propagation. In this method of STAT-ART, a diagnostic CT scan or even a diagnostic MR scan can be utilized as the primary dataset of the STAT-ART pre-plan. The deformation of the CT scan to the MR scan in MRgoART is eliminated; advantageous when large anatomical mismatches are anticipated and therefore eliminates the need and time to perform manual density corrections. During the online adaptive process with a single bulk density override, the external contour is simply defined based on the treatment MR scan, enabling an efficient and robust method of density propagation.

#### Deformation of Diagnostic CT to MR of the Day

An alternative to density override(s) is to utilize electron density information obtained from a diagnostic CT scan. In the MRgoART workflow of the MRIdian, the pre-plan primary dataset of the diagnostic CT scan is deformably registered to the frame of reference of the treatment MR scan utilizing an inverse-consistent, free-form multi-modality DIR with a similarity metric of mutual information and regularization proportional to the Jacobian of the deformation vector field [Mittauer et al. (under review)].

Beyond anatomical setup differences that may require manual density corrections, additional deformation challenges may include a limited field of view on the diagnostic CT scan with missing tissue information. When density corrections are necessary during the time of adaptation, an available override contour of air, bone, and/or soft tissue may be utilized to enable manual electron density edits. It is recommended to input such empty contours with pre-defined electron density of respective air, bone, and soft tissue to the pre-plan to enable the approach of manual electron density edits during online adaptation.

#### Deformation of Template CT to MR of the Day

When prior diagnostic imaging is not readily accessible, a template plan based on the anatomical site of interest (i.e., pelvis, abdomen, thorax, extremity, etc.) can be adapted to the patient's setup at the time of treatment. The alternative method of utilizing a template plan eliminates the time for pre-plan generation, and may be most applicable for urgent palliative cases providing machine availability. The deformation workflow of the CT scan to the treatment MR scan remains the same as the above method, with larger potential for density corrections to be required.

## Discussion

### Adoption of Hypofractionated Palliative Care Toward Single Fraction

In a recent review of palliative radiotherapy, Rich et al. found the adoption of a single fraction course underutilized compared to conventional fractionated course (21), even with recent recommendations (22–24) emphasizing a single-fraction or short-course palliative care. The movement toward a single fraction course for palliative radiotherapy enables two key benefits: cost-reduction and patient time and convenience (21, 25–27). As patients with oligometastatic disease live longer, the need for a more sophisticated, short-course planning and delivery approach is evident over historical parallel-opposed techniques of 30 Gy in 10 fractions.

With MR linacs becoming more common place (28), the adoption of a single fraction course with MR-guidance is a viable approach. The STAT-ART workflow leverages existing volumetric imaging from radiology, eliminating an additional simulation CT. The STAT-ART technique utilizes routine clinical MRgRT workflows, i.e., adaptive radiotherapy. Moreover, MR-guidance enables greater confidence based on real-time image guidance and greater dose conformality, supporting a single fraction approach. A randomized trial comparing efficacy and toxicity between single fraction palliative care on CT-based IGRT vs. MR-based IGRT has yet to be conducted. While several randomized single fraction vs. multiple fraction trials have been carried out, the concerns of toxicities and efficacy of the 8 Gy in 1 fraction regimen still remain. Shuja et al. and Howell et al. described utilization of 3–5 cm margin and <2 cm margin, respectively, from the radiographic involvement for single fraction palliative radiotherapy on conventional c-arm linac modalities (29, 30). The superior bony metastasis visualization combined with improved soft tissue visualization allow for greater precision and enable the utility of a reduced margin in MRgRT of 0.3–0.5 cm as previously demonstrated by Mittauer et al. (16). Howell et al. specifically cited radiation oncologists' concerns of gastrointestinal (GI) toxicities associated with 8 Gy in a single fraction (30). The online adaptive capabilities, to visualize adjacent organs at risk (OARs) and modify dose based on neighboring GI OARs, combined with a smaller margin required due to the reduction in setup uncertainty enable MR-guided single fraction radiotherapy a clear benefit.

Furthermore, to manage the increase in the number of oligometastatic patients, reducing the number of treatments to fewer fractions could potentially lessen the overall burden on hospitals, and ultimately reduce the number of machines required per patient population.

### STAT-ART at the University of Wisconsin-Madison

Our STAT ART program at the University of Wisconsin-Madison was implemented in October 2015 on the MRIdian cobalt, and since then transitioned to the MRIdian linac. Our initial experiences of the STAT-ART program have been briefly reported by Hill et al. (31) and De Costa et al. (32), and includes a retrospective review of the first 18 patients treated with STAT-ART from October 2015 to November 2016.

The indication for STAT-ART included patients with metastatic cancer presenting with pain, obstruction, and bleeding. The majority of STAT-ART patients were treated with a prescription of 8 Gy in a single fraction. STAT-ART planning and treatment delivery was typically >30 min between the patient entering and exiting the treatment vault, compared with a mean time from CT simulation to delivery of first treatment of 29.5 h (95% CI, 23.7–35.2) for a similar sample of urgent palliative cases planned and treated with the conventional radiotherapy workflow. The median delivery time of STAT-ART was 122 s (*N* = 18 patients).

Excellent clinical outcomes were observed and were in line with historical and sampled controls: pain reduction in 11 of 14 patients, improvement of obstructive symptoms in 3 of 3 patients, and hemostasis in 1 of 1 patient. Overall, physician and patient response to the program has been positive, as plan quality has improved while time commitments have been comparable to or less than a conventional simulation-and-treatment workflow. Future efforts include characterizing the dose difference to organs at risk and conformality metrics between STAT-ART plans and conventional parallel-opposed beam geometries.

### MR-Based Planning for Bony Metastases

MR-based treatment planning offers better soft tissue contrast for target delineation as compared to CT simulation (16). However, one particularly interesting finding of the STAT-ART program has been the ability of the MRIdian TrueFISP MR sequences used for treatment planning to identify contrast other than in soft tissues, as shown in Figure 2 for bony metastases. Because pre-plan contours are routinely updated to encompass disease identified on the treatment planning MR, the ability to target disease in bone has been invaluable.

### Additional Workflow Advantages

Through performing treatment planning, contouring, image registration, and treatment delivery on a single platform such as the MRIdian system not only can allow for improvement in clinical efficiency, but also possibly allow for the decrease in the clinical errors from the use of multiple modalities and planning systems. The MRIdian system eliminates the time and need for treatment planning by dosimetry when utilizing template plans. Furthermore, simulation is done in the true treatment position on MRIdian, an advantage over conventional linac-based RT, enabling both enhanced contrast for target delineation and reduction of setup uncertainties.

### Dose Calculation and Deformation Considerations

There are limitations in dose calculation accuracy of STAT-ART when performing bulk density overrides or using deformed diagnostic CT data. For example, with a bulk density override of water for patient anatomy of the pelvis and abdomen, the calculation error is likely on the order of 2%; as demonstrated by Lee et al. for pelvis with absolute dose differences ranging 0–5% inside the planning target volume for uniform density override of water compared to dose calculated on the respective CT scan (33). Larger magnitude of errors would present for other anatomical sites such as thorax/lung. Here, deformation of the diagnostic CT to the treatment MR would be more appropriate.

Electron density propagation of the diagnostic CT to the treatment MR presents has additional uncertainties. The image value to density conversion may not be characterized for the energy spectrum and/or applicable CT scanner of the diagnostic CT dataset at hand. While it may be feasible to characterize all CT scanners in an institution's radiology department, the body of work would be non-trivial and not inclusive of scanners from outside institutions for patients referred for treatment. A potentially more practical approach would be to incorporate CT energy-dependent image value to density table (IVDT) curves, as inter-scanner dependences are minimal to image value variation and on the order of acceptable dose calculation uncertainties for palliative care. A phantom with a range density inserts can be utilized to quantify the Hounsfield unit values as a function of CT energy. Repeat monoenergetic CT scans over an energy range would be acquired to benchmark the IVDT dependent curves. During initial planning the user would then select the respective IVDT curve based on the DICOM tag of the patient's diagnostic CT.

A limitation of the fidelity of the propagation of electron density is the overall voxel resolution. Partial-voxel effects can influence the deformable image registration quality as can be propagated from the initial diagnostic CT scan and/or in the resultant deformed CT, resampled in the resolution and frame of reference of the treatment planning MR. The user is recommended to note the influence partial-voxel effects on deformed electron density accuracy and the potential impact on dose calculation accuracy.

Another challenge of the deformable image registration of the diagnostic CT to the treatment MR is the large anatomical differences between the scans. Figure 2 highlights the posterior anatomical deformations between the curved tabletop of the diagnostic CT and the flat tabletop of the radiotherapy system. Additional deformation differences may include patient arm position or even missing tissue due to limited field of view on the diagnostic CT scan. All of these require review of the CT-MR deformation and may require additional effort and time during the online adaptive workflow to manually correct the electron density using segmentation and overrides.

### Challenges to Implementation

The STAT-ART program relies on capabilities of MRgoART. This work has been presented based on the platform of the MRIdian system as has been implemented at our institution. Modified practice of STAT-ART for other systems with CT/MR on rails or other IGRT systems can be employed. The work to commission and to implement the deformable image registration and dose calculation of MRgoART has been previously described [Mittauer et al. (under review)].

There is some potential for errors to occur in the MRgoART workflow since the plan is adapted on the fly. However, for clinical MRgRT users, MRgoART has become a routine part of everyday workflow (34). The MRgoART utilizes a secondary calculation-based QA to verify plan fidelity. For 3D conformal plans this follows conventional radiotherapy workflow as more sophisticated measurement-based QA are not necessary if the beam model has been appropriately characterized and validated. Secondly, calculation-based methods have been previously shown to be in line with measured-based QA for the MRgoART process (17).

Another unique consideration when implementing MRgoART and STAT-ART is the overall time the patient is on the table in the treatment position. While the STAT-ART process rapidly decreases the time from consultation to treatment for these urgent palliative cases, the “table time” may be longer due to the adaption process. Some patients may not tolerate the 20–30 min on the treatment table as required for the STAT-ART process due to symptomatic pain.

### Alternative Rapid Palliative RT on TomoTherapy

The University of Virginia have implemented a rapid palliative radiotherapy technique using CT-based IGRT of TomoTherapy (Accuracy Inc, Madison, WI) with their “STAT RAD” program (2, 3). The STAT RAD workflow utilizes the on-board MVCT capabilities of TomoTherapy to simulate the patient in treatment position followed by rapid treatment plan generation, quality assurance of the plan with exit dosimetry through the on-board CT detector, and treatment delivery. Since the treatment planning capabilities are not integrated into the simulation and delivery console, plan generation is performed on a separate work station, eliminating an online adaptive approach. The University of Virginia has successfully piloted the program with 50 patient treats reported to date in 2012 (3).

## Conclusion

The integration of a simulator, treatment planning system, and delivery system into a single platform enables the opportunity of STAT-ART, a rapid-access treatment for patients presenting with urgent palliative needs. Electron density information for MR-based planning of STAT-ART without formal CT simulation can be incorporated with either a bulk density override or deformable image registration of diagnostic CT to the treatment MR. The online adaptive features of STAT-ART enable adaptation of a preexisting pre-plan or template plan, reducing the time pressure for urgent palliative radiotherapy. Another key advantage of MRgoART is the superior plan and treatment quality as real-time plan adaptation is performed to the anatomy at treatment compared to the simulation day anatomy as performed in conventional radiotherapy. STAT-ART has great potential in the management of the palliative radiotherapy, making efficient use of both staffing time and resources and expediting palliative care with similarly successful clinical outcomes.

## Data Availability Statement

The raw data supporting the conclusions of this manuscript will be made available by the authors, without undue reservation, to any qualified researcher.

## Ethics Statement

The studies involving human participants were reviewed and approved by University of Wisconsin, Institutional Review Board. Written informed consent for participation was not required for this study in accordance with the national legislation and the institutional requirements.

## Author Contributions

KM, PH, MG, MB, and JB designed the workflow. PH and A-MD performed the data summary. KM drafted the manuscript. KM, PH, MG, A-MD, RK, MB, and JB iteratively revised the manuscript.

### Conflict of Interest

KM reports personal fees from ViewRay Inc. and ownership in MR Guidance, LLC; MB reports personal fees from ViewRay Inc.; JB reports membership of Advisory Board of ViewRay Inc. and ownership in MR Guidance, LLC during the conduct of the study. The remaining authors declare that the research was conducted in the absence of any commercial or financial relationships that could be construed as a potential conflict of interest.
